# The effects of locally administered aminophylline in patients undergoing ureteroscopic lithotripsy: a systematic review with exploratory meta-analysis

**DOI:** 10.3389/fruro.2026.1822923

**Published:** 2026-06-17

**Authors:** Mansour Alnazari, Ahmad Badawi, Osama Alamri, Abdullah Alsehli, Omar Alamri, Mohammed Alhusayni, Abdullah Alghamdi, Amr Mahran

**Affiliations:** 1 Department of Urology, College of Medicine, Taibah University, Medina, Saudi Arabia; 2 College of Medicine, Taibah University, Medina, Saudi Arabia; 3 Urology, King Salman bin Abdulaziz Medical City, Medina, Saudi Arabia; 4 Department of Urology, King Faisal Specialist Hospital and Research Centre, Medina, Saudi Arabia; 5 Department of Urology, Faculty of Medicine, Assiut University, Assiut, Egypt

**Keywords:** aminophylline, lithotripsy, meta-analysis, systematic review, ureteroscopy, urolithiasis

## Abstract

**Purpose:**

Aminophylline, a methylxanthine derivative, acts as a smooth muscle relaxant through non-competitive inhibition of phosphodiesterase. It has been suggested to facilitate ureteroscopic lithotripsy by relaxing ureteral smooth muscle. The aim of this systematic review is to assess the available evidence on locally administered aminophylline in patients undergoing ureteroscopic lithotripsy and, where feasible, to perform an exploratory meta-analysis to estimate pooled effects.

**Methods:**

A systematic search was conducted through November 2025. Three randomized controlled trials (RCTs) met the inclusion criteria. The primary outcome was residual stone proportions (derived from the stone-free rate). Secondary outcomes included operative time, postoperative ureteral stenting, and auxiliary procedures. The risk of bias was assessed using the Cochrane Risk of Bias 2 (RoB-2) tool. This review was registered with PROSPERO (CRD420251229352).

**Results:**

Three RCTs enrolling 310 patients (155 receiving aminophylline and 155 receiving placebo) were included. Local aminophylline was associated with significantly fewer residual stones (RR 0.3; 95% CI: 0.13, 0.69; *p* = 0.025; I² = 0%) and fewer auxiliary procedures (RR 0.15; 95% CI: 0.06, 0.38; *p <*0.001; I² = 0%). Reductions in operative time and postoperative ureteral stenting were observed in the primary analysis; however, these did not remain statistically significant after applying the Hartung–Knapp–Sidik–Jonkman (HKSJ) correction.

**Conclusion:**

This exploratory meta-analysis provides preliminary evidence that locally administered aminophylline may reduce residual stone fragments and auxiliary procedures during ureteroscopic lithotripsy. Reductions in operative time and ureteral stenting did not retain statistical significance in sensitivity analyses and should be considered hypothesis-generating. These findings should be interpreted with caution, given the low certainty of the evidence. Adequately powered randomized trials are required to validate these results.

**Systematic review registration:**

https://www.crd.york.ac.uk/prospero/, identifier CRD420251229352.

## 1 Introduction

Urolithiasis is a prevalent chronic condition with a substantial global health burden. A systematic analysis of the Global Burden of Disease Study (2000–2021) estimated approximately 106 million incident cases worldwide in 2021, with males accounting for 67% of cases (1), while the global prevalence was estimated at approximately 10.9% of the adult population (2). Approximately 25% of patients with renal stones will require intervention (3).

Ureteroscopy (URS) with laser lithotripsy is a well-established minimally invasive approach for the management of ureteric and renal stones (4). However, limited ureteral dilatation or ureteral spasms may occasionally impede ureteroscope advancement, potentially increasing the rate of secondary procedures, prolonging operative time, and contributing to overall treatment costs. These intraoperative challenges have prompted investigation into pharmacological adjuncts that facilitate ureteroscope passage by relaxing ureteral smooth muscle.

Methylxanthines are purine-derivative agents that induce smooth muscle relaxation. Their effects are primarily mediated through non-competitive inhibition of phosphodiesterase (PDE), leading to increased intracellular cyclic adenosine monophosphate (cAMP) and cyclic guanosine monophosphate (cGMP), resulting in smooth muscle relaxation (5, 6). Preclinical evidence has demonstrated that theophylline and selective PDE-IV inhibitors induce measurable relaxation of the human and animal ureter (7), providing a mechanistic foundation for their intraoperative use. Aminophylline, a soluble salt of theophylline, has been investigated as a locally administered adjunct during URS to exploit these smooth muscle relaxant properties (8). Local administration offers a theoretical advantage over the systemic route by delivering the drug directly to the ureteral mucosa while minimizing systemic side effects.

Several other pharmacological adjuncts have been investigated to facilitate ureteroscopy, including alpha-1 adrenergic blockers such as tamsulosin (9, 10) and silodosin (11, 12), calcium channel blockers such as nifedipine (13), and, more recently, phosphodiesterase-5 inhibitors (12, 14). While the evidence for alpha-blockers has been the subject of several systematic reviews and meta-analyses (10), the evidence regarding locally administered methylxanthines during ureteroscopic lithotripsy remains limited and has not been systematically synthesized. To our knowledge, no prior systematic review has evaluated this specific intervention. The aim of this systematic review is to evaluate the available evidence on the effects of locally administered aminophylline on procedural and clinical outcomes in patients undergoing ureteroscopic lithotripsy and, where feasible, to perform an exploratory meta-analysis to estimate pooled effects.

## 2 Methods

This systematic review with exploratory meta-analysis was conducted in accordance with the Preferred Reporting Items for Systematic Reviews and Meta-Analyses (PRISMA) guidelines (15) and was registered in the International Prospective Register of Systematic Reviews (PROSPERO) under protocol number CRD420251229352. The study outcomes were assessed using the Grading of Recommendations, Assessment, Development and Evaluation (GRADE) tool (https://gdt.gradepro.org/app/) (16).

### 2.1 Search strategy and study selection

**Figure 1:**
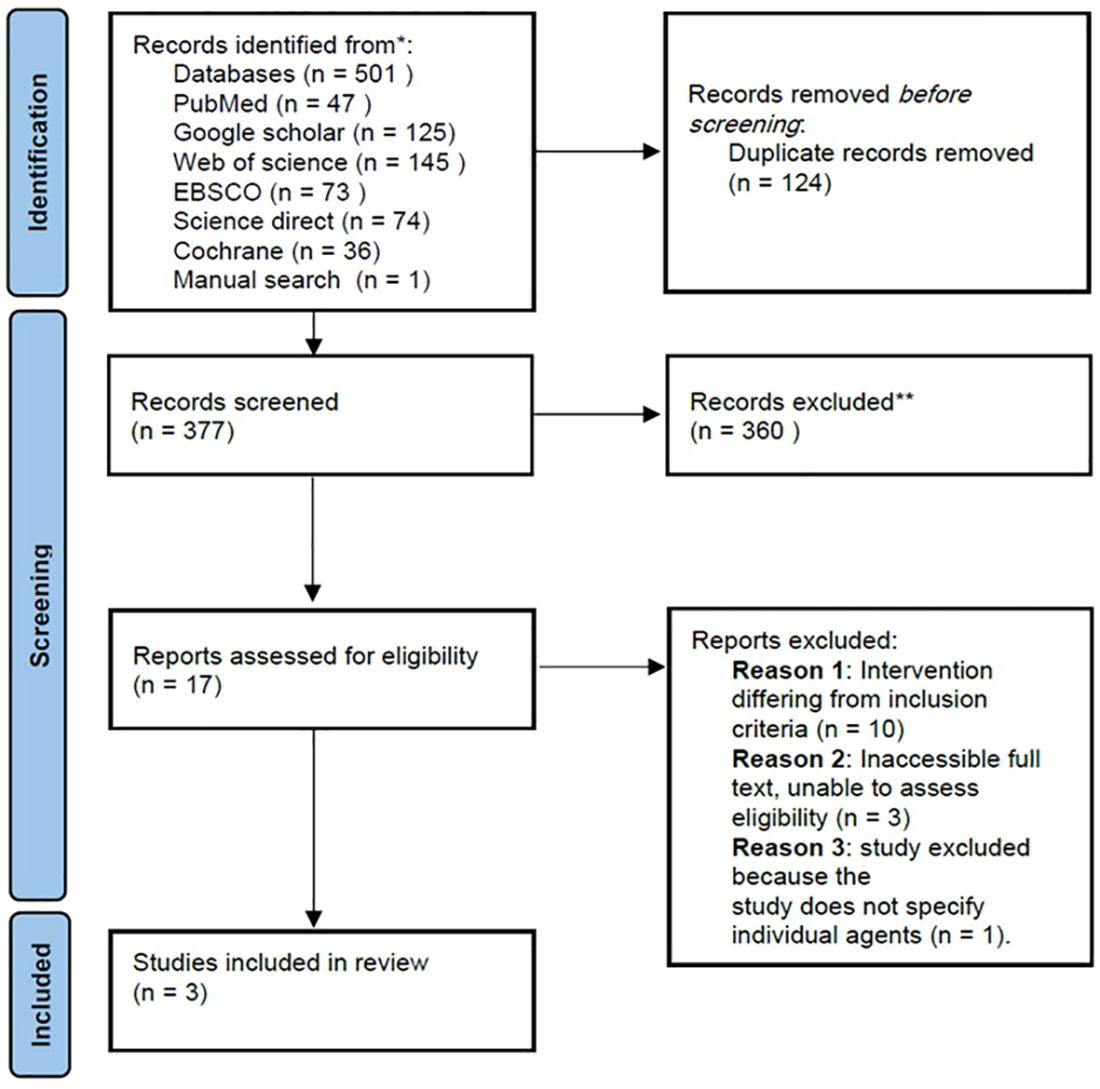
PRISMA flowchart.

We systematically searched PubMed (MEDLINE), Google Scholar, ScienceDirect, EBSCO, Web of Science, and Cochrane through November 2025. Keywords used included “ureteroscopy,” “lithotripsy,” “stone removal,” “kidney stone,” “renal stone,” “ureteral stone,” “theophylline,” “aminophylline,” and “methylxanthines.” The detailed systematic search strategy is available in **Supplementary File 1**. A manual search for additional relevant studies using references from retrieved articles was also performed. For articles in which the full text was not available, we contacted the corresponding authors; however, no responses were received. Two reviewers (AALS and OSA) independently performed the screening. The results of the literature search were imported into Rayyan software (17), and duplicates were removed. All identified articles were systematically assessed using the inclusion and exclusion criteria. The references of the included studies were screened using snowballing to identify additional studies. Disagreements were resolved by a third author (AB). A PRISMA flowchart is shown in **Figure 1**.

### 2.2 Eligibility criteria

We included peer-reviewed randomized controlled trials, regardless of language, that evaluated local administration of aminophylline during ureteroscopic lithotripsy compared with placebo in adult patients undergoing ureteroscopy for ureteral or renal stones. Only studies reporting at least one relevant procedural or clinical outcome of interest were included.

### 2.3 Data extraction

For each included study, we extracted data pertaining to study identification (first author, publication year, country, and study design), patient characteristics (total number of patients in the intervention and control groups and patient demographics), stone-related characteristics (stone size, stone-free rates, and residual stones), procedural information (dose of aminophylline, operative time, and ureteral stenting), any reported complications, and finally, the incidence of auxiliary procedures (secondary ureteroscopy or extracorporeal shock wave lithotripsy “ESWL”).

### 2.4 Data analysis

The primary outcome was the rate of residual stones (calculated from the stone-free rate). It should be noted that stone-free definitions, imaging modality, post-operative assessment timing, and residual fragment size thresholds were not uniformly standardized across the included trials. This heterogeneity represents a potential source of measurement variability and limits direct comparability of the primary endpoint across studies. Secondary outcomes included the need for postoperative ureteral stenting, operative time, and auxiliary procedures. Residual stone proportions, postoperative ureteral stenting, and auxiliary procedures were analyzed using the number of events and the total number of patients in the aminophylline (intervention) and placebo groups. Risk ratios (RR) and their 95% confidence intervals (CI) were calculated. Operative time was analyzed using the means and standard deviations (SD) in each group, and effect sizes were calculated as standardized mean differences (SMD) with 95% confidence intervals. Absolute SMDs were interpreted as a small effect if <0.2, a medium effect if between 0.2 and 0.8, and a large effect if >0.8. Barzegarnezhad et al. (8) reported an mean operative time of 4.2 min as compared with 33 min–54 min in the other trials, likely reflecting measurement of ureteroscope insertion time rather than total procedure time; therefore, it was excluded from this analysis. The I² statistic was used to assess the degree of heterogeneity among the included studies for each outcome. Low heterogeneity was defined as 0%–30%, moderate as 30%–60% and substantial as >60%. Random-effects model with Restricted Maximum Likelihood (REML) was employed in all analyses to mitigate the effects of heterogeneity. It should be noted that with only two contributing studies for operative time and ureteral stenting, the I² estimate is unreliable and cannot meaningfully quantify heterogeneity. Tau² could also be unreliable with few studies. These statistics were reported for the purpose of descriptive completeness. The analysis was initially performed without the Hartung, Knapp, Sidik, and Jonkman (HKSJ) method for outcomes with increased heterogeneity, and a second sensitivity analysis using the HKSJ method was then employed. Given the small number of included studies (n = 3), formal assessment of publication bias (e.g., funnel plot) was not feasible; however, publication bias cannot be excluded as a potential limitation. Package “meta” (18) was used to generate comparative analyses as part of R software (19) version 4.5.0.

### 2.5 Local administration techniques

Intervention group: In the studies by Barzegarnezhad et al. and Khan et al. (8, 20), aminophylline 250 mg/10 mL, mixed with normal saline, was used to prepare a total of 150 mL irrigation solution, which was instilled via the ureteroscope after bladder emptying using a Foley’s catheter. For the study by Lubana et al. (21), aminophylline 10 mL, mixed with normal saline, was utilized to make a total of 150 mL solution, which was delivered via the ureteroscope.

Control group: received local normal saline in all three included studies.

Waiting time: Ureteroscopy and lithotripsy were performed 5 min after irrigation in all three included studies.

### 2.6 Risk of bias assessment

**Figure 2:**
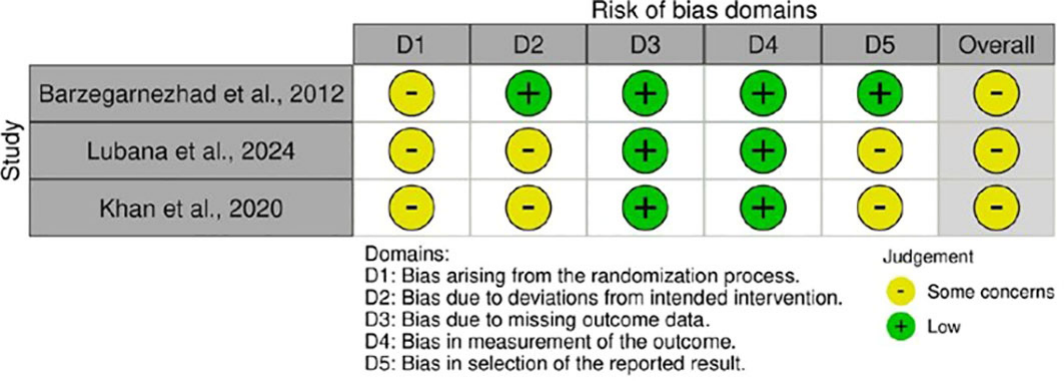
Cochran risk of bias 2 (RoB2) tool evaluation of the included randomized trials.

The risk of bias assessment was conducted using the Cochrane’s Risk of Bias 2 (RoB 2) tool for randomized controlled trials. The three studies were rated as having low risk of bias to “some concerns” of bias. The risk of bias is shown in **Figure 2**.

### 2.7 Data synthesis

A mixed-methods approach was used, which entailed the use of both quantitative and qualitative data analyses. Given the small number of included studies (n = 3), the quantitative approach was performed as an exploratory meta-analysis to provide preliminary pooled estimates and to identify the direction and consistency of effects across trials, rather than to generate definitive effect sizes. This was feasible because all the included studies addressed the same clinical question, had comparable patient populations, and had comparable intervention protocols (22). To mitigate the risk of misleading precision inherent in small meta-analyses, we employed a random-effects model with restricted maximum likelihood (REML) estimation throughout and performed sensitivity analyses using the Hartung–Knapp–Sidik–Jonkman (HKSJ) adjustment, which produces wider and more conservative confidence intervals when the number of pooled studies is small. Outcomes that were reported inconsistently across studies, including stone size and complications, were synthesized qualitatively. This allowed for a comprehensive approach to data interpretation by including all available evidence.

## 3 Results

### 3.1 Study selection and characteristics

Our initial search yielded 501 records across all databases. After removing 123 duplicate entries and excluding ineligible studies based on title and abstract, a total of 17 full-text articles were assessed for eligibility. Of these, three randomized controlled trials (8, 20, 21) met the prespecified inclusion criteria and were included in the final review (**Figure 1**). Across the included trials, a total of 310 patients were enrolled, of whom 155 patients (50%) received aminophylline and 155 patients (50%) received a placebo. The mean age of participants across the studies ranged from 34.8 to 47.6 years. Stone size was reported in only one study, where the intervention group had a mean size of 5.4 mm ± 3.1 mm compared to 5.7 mm ± 4.02 mm in the control group (8). The characteristics of the included studies are summarized in **Table 1**.

**Table 1:** Baseline characteristics of included studies. .

Author (year)	Country	Study design	Sample size	Mean age, SD	Male (%)	Dose/frequency	Stone free rate	Operative time	Ureteral stenting	Auxiliary procedures	Complications
Barzegarnezhad et al. (2012) (8)	Iran	Double-blind randomized clinical trial	I = 60 patients C = 60 patients	I = 34.8 ± 13.2 C = 35.4 ± 12.7	66.70	I = 250 mg C = 150 ml Once	I = 95% C = 71.60%	I = 4.2 ± 2.61 C = 8.4 ± 2.9	I = 23.3% C = 73.3%	ESWL I = 3 (5%) C = 18 (30%)	No significant post-procedural complications. No significant increase in hypertension or tachycardia in the intervention group
Lubana et al. (2024) (21)	India	Prospective, Randomized Clinical Trial	I = 50 patients C = 50 patients	I = 38.5 ± 9.51 C = 40.46 ± 9.49	NR	I = 250 mg C = 150 ml Once	I = 82% C = 44%	I = 33.54 ± 4.33 C = 54.56 ± 15.90	I = 38% C = 68%	ESWL/secondary ureteroscopy I = 2 (4%) C = 15 (30%)	No significant post-procedural complications. No significant increase in febrile UTIs, hypertension or tachycardia in the intervention group
Khan et al. (2020) (20)	Pakistan	Randomized Clinical Trial	I = 45 patients C = 45 patients	I = 47.56 ± 18.1	54.40	I = 250 mg C = 150 ml Once	I = 88.88% C = 71.11%	I = 39.96 ± 6.99 C = 48.71 ± 6.95	NR	NR	NR

SD, standard deviation; I, intervention; C, control; UTI, urinary tract infection; NR, not reported.

### 3.2 Efficacy outcomes

#### 3.2.1 Primary analyses

##### 3.2.1.1 Residual stones

**Figure 3:**
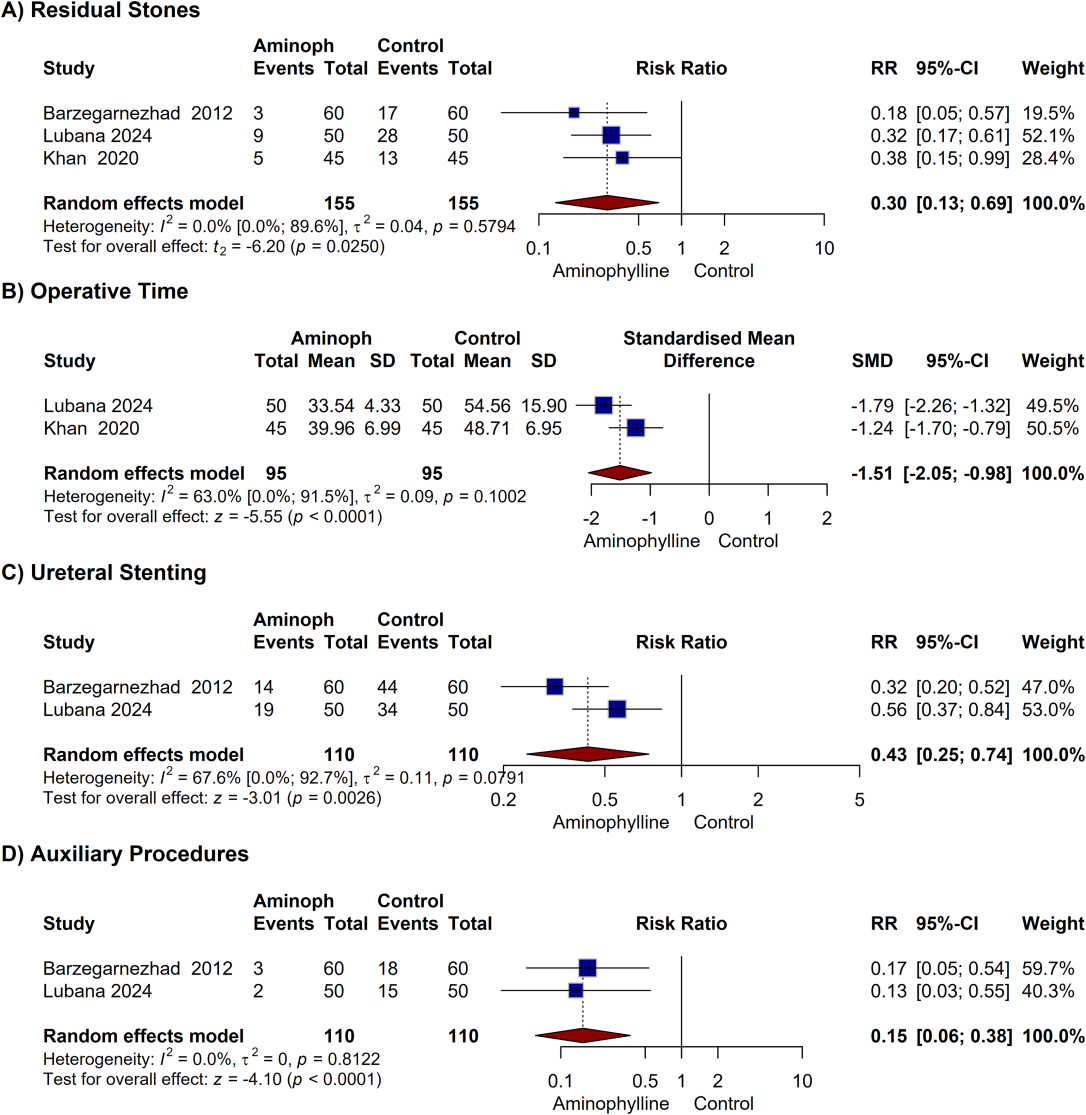
Forest plots for **(A)** Residual Stones, **(B)** Operative time, **(C)** Ureteral stenting, and **(D)** Auxiliary procedures. SD, standard deviations; CI, confidence intervals; SMD, standardized mean difference; RR, risk ratio; Aminoph, aminophylline.

Three studies (8, 20, 21) enrolling 310 patients (155 in the aminophylline group and 155 in the control group) were included. The aminophylline group was associated with a significant reduction in residual stones (RR 0.30; 95% CI: 0.13, 0.69; *p* = 0.025). There was no heterogeneity among the included studies (I^2^ = 0.0% (0.0%, 89.6%); *p* = 0.58) (**Figure 3A**).

##### 3.2.1.2 Operative time

###### 3.2.1.2.1 Without HKSJ method

Only two studies (20, 21) enrolling 190 patients (95 in the aminophylline group and 95 in the control group) were included. The aminophylline group was associated with significantly reduced operative time (large effect; SMD −1.51; 95% CI −2.05, −0.98; *p <*0.001). The heterogeneity among the included studies was substantial (I^2^ = 63% (0.0%, 91.5%); *p* = 0.1) (**Figure 3B**).

##### 3.2.1.3 Ureteral stenting

###### 3.2.1.3.1 Without HKSJ method

Only two studies (8, 21) enrolling 220 patients (110 in the aminophylline group and 110 in the control group) were included. The aminophylline group was associated with significantly reduced ureteral stenting (RR 0.43; 95% CI: 0.25, 0.74; *p* = 0.003). The heterogeneity among the included studies was substantial (I^2^ = 67.6% (0.0%, 92.7%); *p* = 0.08) (**Figure 3C**).

#### 3.2.2 Auxiliary procedures

Only two studies (8, 21) enrolling 220 patients (110 in the aminophylline group and 110 in the control group) were included. The aminophylline group was associated with significantly fewer auxiliary procedures (RR 0.15; 95% CI: 0.06, 0.38; *p <*0.001). There was no heterogeneity among the included studies (I^2^ = 0.0%; *p* = 0.81) (**Figure 3D**).

### 3.3 Sensitivity analyses

#### 3.3.1 Ureteral stenting

##### 3.3.1.1 With HKSJ method

**Figure 4:**
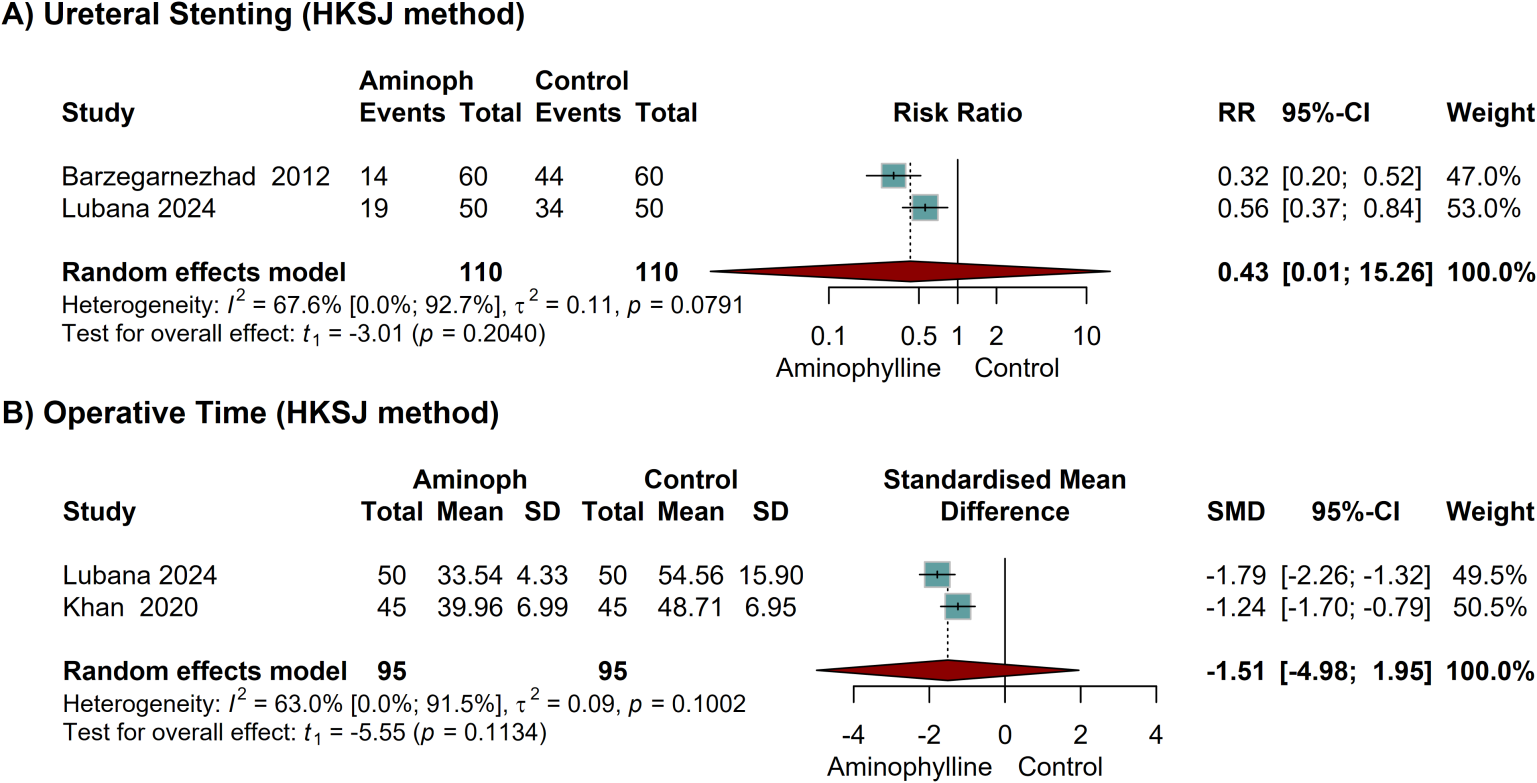
Sensitivity forest plots for **(A)** Ureteral stenting and **(B)** Operative time with HKSJ method. SD, standard deviations; CI, confidence intervals; SMD, standardized mean difference; RR, risk ratio; Aminoph, aminophylline.

Only two studies (8, 21) enrolling 220 patients (110 in the aminophylline group and 110 in the control group) were included. No statistically significant difference was observed between the studied groups (RR 0.43; 95% CI: 0.01, 15.26; *p* = 0.2). The heterogeneity among the included studies was substantial (I^2^ = 67.6% (0.0%, 92.7%); *p* = 0.08) (**Figure 4A**).

#### 3.3.2 Operative time

##### 3.3.2.1 With HKSJ method

Only two studies (20, 21) enrolling 190 patients (95 in the aminophylline group and 95 in the control group) were included. No statistically significant difference was observed between the studied groups (SMD −1.51; 95% CI −4.98, 1.95; *p* = 0.11). The heterogeneity among the included studies was substantial (I^2^ = 63% (0.0%, 91.5%); *p* = 0.1) (**Figure 4B**).

### 3.4 Safety outcomes

Regarding safety outcomes, aminophylline was generally well tolerated in all included trials. No significant complications were reported. Local aminophylline did not increase the risk of febrile urinary tract infections, hypertension, or tachycardia (8, 21).

### 3.5 GRADE certainty of evidence

Using the GRADEpro tool, the outcomes of the current exploratory meta-analysis were rated as very low to low certainty of evidence. The primary analyses of residual stones and auxiliary procedures showed low certainty, while the primary and HKSJ-corrected analyses of the operative time and ureteral stenting rates demonstrated very low certainty of evidence (**Table 2**).

**Table 2:** GRADE certainty of evidence for the study outcomes.

Certainty assessment								Summary of findings				
Outcome	# Studies	Study design	Risk of bias	Inconsistency	Indirectness	Imprecision	Publication bias	No. of patients			Effect	Certainty
								Aminoph	Control	Relative (95% CI)	Absolute (95% CI)	
Residual stones	3	RCT	Serious	Not Serious I2: 0%	Not serious	Serious	Undetected	17/155 (11%)	58/155 (37%)	RR 0.3 (0.13, 0.69)		⊕○○○ LOW
Operative time	2	RCT	Serious	Serious I2: 63%	Not serious	Serious	Undetected	95	95		SMD 1.51 SD lower (2.05 lower to 0.98 lower)	⊕○○○ VERY LOW
Ureteral stenting	2	RCT	Serious	Serious I2: 67.6%	Not serious	Serious	Undetected	33/110 (21%)	78/110 (71%)	RR 0.43 (0.25, 0.74)		⊕○○○ VERY LOW
Auxiliary procedures	2	RCT	Serious	Not Serious I2: 0%	Not serious	Serious	Undetected	5/110 (4.5%)	33/110 (30%)	RR 0.15 (06, 0.38)		⊕○○○ LOW
Operative time (HKSJ-corrected)	2	RCT	Serious	Serious I2: 63%	Not serious	Serious	Undetected	95	95		SMD 1.51 SD lower (4.98 lower to 1.95 higher)	⊕○○○ VERY LOW
Ureteral stenting (HKSJ-corrected)	2	RCT	Serious	Serious I2: 67.6%	Not serious	Very serious	Undetected	33/110 (21%)	78/110 (71%)	RR 0.43 (0.1, 15.6)		⊕○○○ VERY LOW

^*^ Aminoph, Aminophylline, HKSJ-corrected confidence intervals shown for operative time and ureteral stenting; CI, confidence interval; OR, odds ratio; SMD, standardized mean difference.

Risk of bias: 2/3 trials not double-blind. Inconsistency: I² >60% for operative time and stenting. Imprecision: small sample sizes, wide CIs, Publication bias: not downgraded but cannot be excluded.

## 4 Discussion

To the best of our knowledge, this systematic review with exploratory meta-analysis is the first to systematically synthesize all available RCT evidence on this topic, providing preliminary evidence that locally administered aminophylline may improve short-term stone clearance during ureteroscopic lithotripsy. The exploratory pooled analysis yielded a 70% reduction in the risk of residual stones (RR 0.30; 95% CI: 0.13–0.69) and an 85% reduction in the risk of requiring auxiliary procedures (RR 0.15; 95% CI: 0.06–0.38). These figures are derived from three and two small RCTs, respectively, and must be interpreted in the context of low certainty of evidence. The direction of effect was consistent across all included trials. However, reductions in operative time and postoperative ureteral stenting did not retain statistical significance in the HKSJ sensitivity analysis, which is the more conservative and recommended approach for meta-analyses with few studies (23). These findings should therefore be regarded as hypothesis-generating for these secondary outcomes and are intended to inform the design of future adequately powered confirmatory trials.

The observed benefits are biologically plausible. Local aminophylline inhibits phosphodiesterase enzymes, increasing intracellular cAMP and promoting ureteral smooth muscle relaxation (5, 6). This facilitates ureteroscope insertion, improves visualization and irrigation, and promotes complete stone fragmentation with reduced ureteral spasm. It acts as a chemical ureteral dilator analogous to mechanical balloon dilation but without the associated wall stress and mucosal trauma. The improvements in stone-free rates and reductions in auxiliary procedures observed across the included trials are likely a consequence of improved initial ureteroscope access, enabling better stone visualization, more effective lithotripter energy delivery, and improved fragment retrieval. Regarding safety, Barzegarnezhad et al. (8), the only double-blind trial included, reported no aminophylline-attributable complications such as tachycardia or hypotension in 120 patients, suggesting that the local route may avoid the systemic toxicity profile associated with intravenous administration. Shabayek et al. (24) found local aminophylline safe and effective compared with balloon dilation in reducing intraureteral pressure and postoperative complications, while Ghadian et al. (25) and Rehab et al. (26) demonstrated benefits of intravenous aminophylline in reducing post-ureteroscopic complications and postoperative pain, respectively. Collectively, these findings from multiple administration routes and comparators support the biological plausibility of aminophylline’s effects on the ureter during endoscopic procedures.

These findings should be contextualized within the broader landscape of pharmacological adjuncts to ureteroscopy. Alpha-1 adrenergic blockers, particularly tamsulosin, have been the most extensively studied agents; a meta-analysis by Alsaikhan et al. (10) demonstrated that preoperative alpha-blockers significantly improved ureteroscope insertion rates and reduced complications. Calcium channel blockers such as nifedipine (13) and, more recently, phosphodiesterase-5 inhibitors (14) have also shown efficacy in ureteral smooth muscle relaxation. Local aminophylline offers a distinct mechanistic pathway and the advantage of direct topical application. Future head-to-head trials comparing local aminophylline with these agents would help establish its relative efficacy and position in clinical practice.

The principal strengths of this review include its novelty as the first systematic synthesis on this topic, prospective protocol registration, adherence to PRISMA guidelines, the transparent framing of the meta-analysis as exploratory given the limited evidence base, and the use of both standard random-effects and HKSJ sensitivity analyses with transparent reporting of outcomes that lost significance under more conservative methods.

Several limitations must be acknowledged. The small number of included studies (n = 3) with only 310 patients limits statistical power and the precision of pooled estimates; between-study variance may be imprecisely estimated. Only one trial was double-blind, introducing potential performance and detection bias. All three studies were conducted in a limited number of geographic settings (Iran, India, and Pakistan), which may limit generalizability to centers with different surgical equipment, training standards, and patient populations. The primary endpoint (residual stone rate derived from stone-free rate) was subject to variability in definition, imaging modality, and assessment timing across trials, which may have introduced outcome heterogeneity. Furthermore, the considerable heterogeneity in operative time could result from the variation in the measurement (whether including the total procedure time or only the ureteroscope insertion time). Ureteral stenting is also prone to surgeon preferences. Intervention heterogeneity could represent another source of uncertainty. Notably, the aminophylline concentration in Lubana et al. (21) was not reported. Delivery techniques, contact time, and irrigation dynamics were not described adequately. That said, these factors may contribute to between-study variability that extends beyond what standard statistical metrics reflect. To reduce the effects of heterogeneity and imprecision, random-effects modeling with and without the HKSJ correction were employed. It should be noted that in meta-analyses of two to three studies, exclusive reliance on the HKSJ interval may dismiss potentially efficacious interventions when all studies consistently demonstrate benefit (27); conversely, the non-HKSJ results may overstate precision, and both sets of results should be considered together. The pooled RR 0.15 for auxiliary procedures may partly reflect sparse-event inflation rather than the true magnitude of clinical benefit and should be interpreted with caution. Finally, publication bias cannot be excluded, as funnel plot analysis was not feasible.

## 5 Conclusion

This systematic review with exploratory meta-analysis provides preliminary, low-certainty evidence that locally administered aminophylline during ureteroscopic lithotripsy reduces residual stone fragments and the need for auxiliary procedures compared with normal saline, with negligible heterogeneity across the included trials. Reductions in operative time and postoperative ureteral stenting were observed in the primary analysis but did not retain statistical significance after HKSJ correction and should be considered hypothesis-generating. Aminophylline demonstrated an acceptable safety profile with no attributable adverse events reported across the included patients. However, with a total of 310 patients, the current evidence is insufficient to characterize the rare-event safety profile, and larger trials with systematic adverse event reporting are required to further consolidate its safety profile.

These findings should be interpreted with caution given the limited number of included studies, small cumulative sample size, and the lack of double-blinding in two of three trials. The certainty of evidence is low, the pooled estimates are exploratory in nature, and firm clinical recommendations cannot yet be made.

Adequately powered, double-blind, multicenter randomized controlled trials are needed to confirm the observed benefits on stone clearance and auxiliary procedures, clarify the effects on operative time and stent placement with standardized outcome definitions, define the optimal dosing, concentration, and administration technique, and compare local aminophylline directly with other pharmacological adjuncts, including alpha-blockers and calcium channel blockers, to establish its relative efficacy and position in clinical practice.

## Data Availability

The original contributions presented in the study are included in the article/**Supplementary Material**. Further inquiries can be directed to the corresponding author.
