# Supplementary material for: The effects of locally administered aminophylline in patients undergoing ureteroscopic lithotripsy: a systematic review with exploratory meta-analysis
Source: Front Urol. 2026 Jun 17;6:1822923. doi: 10.3389/fruro.2026.1822923 (PMC13318564; doi:10.3389/fruro.2026.1822923)
Supplement: Supplementary file 1 [file SupplementaryFile1.docx]

**Systematic search strategies:**

**PubMed:**

("theophylline"[Title/Abstract] OR "methylxanthine"[Title/Abstract]

OR "theobromine"[Title/Abstract] OR "aminophylline"[Title/Abstract]

OR "paraxanthine"[Title/Abstract])

AND

("ureteroscopy"[Title/Abstract] OR "ureteroscopic lithotripsy"[Title/Abstract]

OR "ureteral stone"[Title/Abstract])

AND

("local instillation"[Title/Abstract] OR "topical"[Title/Abstract]

OR "intraluminal"[Title/Abstract] OR "irrigation"[Title/Abstract])

AND

("clinical outcomes"[Title/Abstract] OR "stone clearance"[Title/Abstract]

OR "operation time"[Title/Abstract] OR "ureteral spasm"[Title/Abstract])

**ScienceDirect:**

("theophylline" OR "methylxanthine" OR "aminophylline")

AND

("ureteroscopy" OR "ureteroscopic lithotripsy")

AND

("local instillation" OR "irrigation")

AND

("clinical outcomes" OR "stone clearance")

**EBSCO:**

(theophylline OR methylxanthine OR aminophylline)

AND

(ureteroscopy OR ureteral calculi OR ureteral stone)

AND

(local instillation OR intraluminal OR irrigation)

AND

(clinical outcomes OR stone clearance OR ureteral spasm)

**Web of Science:**

TS=(theophylline OR methylxanthine OR aminophylline)

AND

TS=(ureteroscopy OR ureteroscopic lithotripsy OR ureteral stone)

AND

TS=(local instillation OR topical OR intraluminal OR irrigation)

AND

TS=(clinical outcomes OR stone clearance OR operation time OR ureteral spasm)
